# Effects of Losartan, Empagliflozin, and Their Combination on Uric Acid Concentrations in People With Type 2 Diabetes

**DOI:** 10.1016/j.xkme.2026.101436

**Published:** 2026-06-13

**Authors:** Danii L.S. Suijk, Preciosa X.C. Simson, Rosalie A. Scholtes, Charlotte M. Mosterd, Anne C. Hesp, Ewout J. Hoorn, Petter Bjornstad, Daniel H. van Raalte

**Affiliations:** 1 Department of Internal Medicine, Diabetes Center, Amsterdam University Medical Center, Amsterdam, the Netherlands; 2 Division of Nephrology and Transplantation, Department of Internal Medicine, Erasmus University Medical Center, Rotterdam, the Netherlands; 3 Department of Pediatrics, University of Washington Medicine Diabetes Institute, University of Washington, Seattle, WA; 4 Department of Medicine, University of Washington Medicine Diabetes Institute, University of Washington, Seattle, WA; 5 Department of Medicine, University of Colorado Anschutz Medical Campus, Aurora, CO

To the Editor:

Increased plasma uric acid (PUA) concentrations are associated with cardiovascular disease and chronic kidney disease in people with type 2 diabetes (Item S1). Recently, approaches have been pursued to reduce PUA concentrations and its associated adverse clinical outcomes (Item S1).

Sodium-glucose transporter 2 (SGLT2) inhibitors have shown to decrease PUA by increasing urinary uric acid excretion, likely mediated through activation of urate transporter 1 (URAT1).^1^, ^2^, ^3^ Importantly, reductions in uric acid were associated with reductions in kidney and cardiovasculardisease after treatment with canagliflozin and empagliflozin.^4^^,^^5^

In addition, renin-angiotensin system inhibitors, in particular losartan, have shown to reduce PUA levels in people with type 2 diabetes and diabetic kidney disease, similar to the effects seen in treatment with SGLT2 inhibitors (Item S1). Losartan may also facilitate urinary uric acid excretion^6^ through URAT1 inhibition. Participants who had lower PUA levels following losartan treatment in the Reduction of Endpoints in NIDDM with the Angiotensin II Antagonist Losartan (RENAAL) trial had reduced risk of cardiovascular outcomes compared with people with unchanged PUA levels (Item S1).

As SGLT2 inhibitors and renin-angiotensin system blockers are often used as combination therapy in people with type 2 diabetes and high-risk of cardiovascular and chronic kidney disease, we aimed to evaluate the separate and combined effects of these drugs on PUA concentrations as well as their urinary clearance.

Twenty-four participants aged between 25 and 75 years were included in this post hoc analyses of the Renohemodynamic Effects of Combined empagliflozin and Losartan (RECOLAR) study and were treated for 1 week with empagliflozin (10 mg), losartan (50 mg), and combination therapy (extended methods, Figure S1; Item S2). Primary endpoints were the effect of treatment on PUA. Participants were predominantly men (88%), were on average 66 ± 6 years old with well-controlled diabetes (HbA1c 7.40% ± 0.90%), and with preserved kidney function (estimated glomerular filtration rate 89 mL/min/1.73 m^2^ [interquartile range 79-90]). Baseline PUA and fractional excretion of uric acid (FeUA) were 5.4 mg/dL (interquartile range 4.9-6.6) and 5.1% (interquartile range 3.6-6.6), respectively. Baseline characteristics are shown in Table S1.

Empagliflozin lowered PUA by 0.84 mg/dL (*P* = 0.009), losartan by 0.44 mg/dL (*P* > 0.99), and the combination therapy by 1.11 mg/dL (*P* < 0.001) (Table S2). Combination therapy had a greater PUA-lowering effect compared with losartan monotherapy (-0.67 mg/dL; *P* < 0.001), but not compared with empagliflozin monotherapy (-0.27 mg/dL; *P* = 0.17) (Fig 1A).

**Figure 1:**
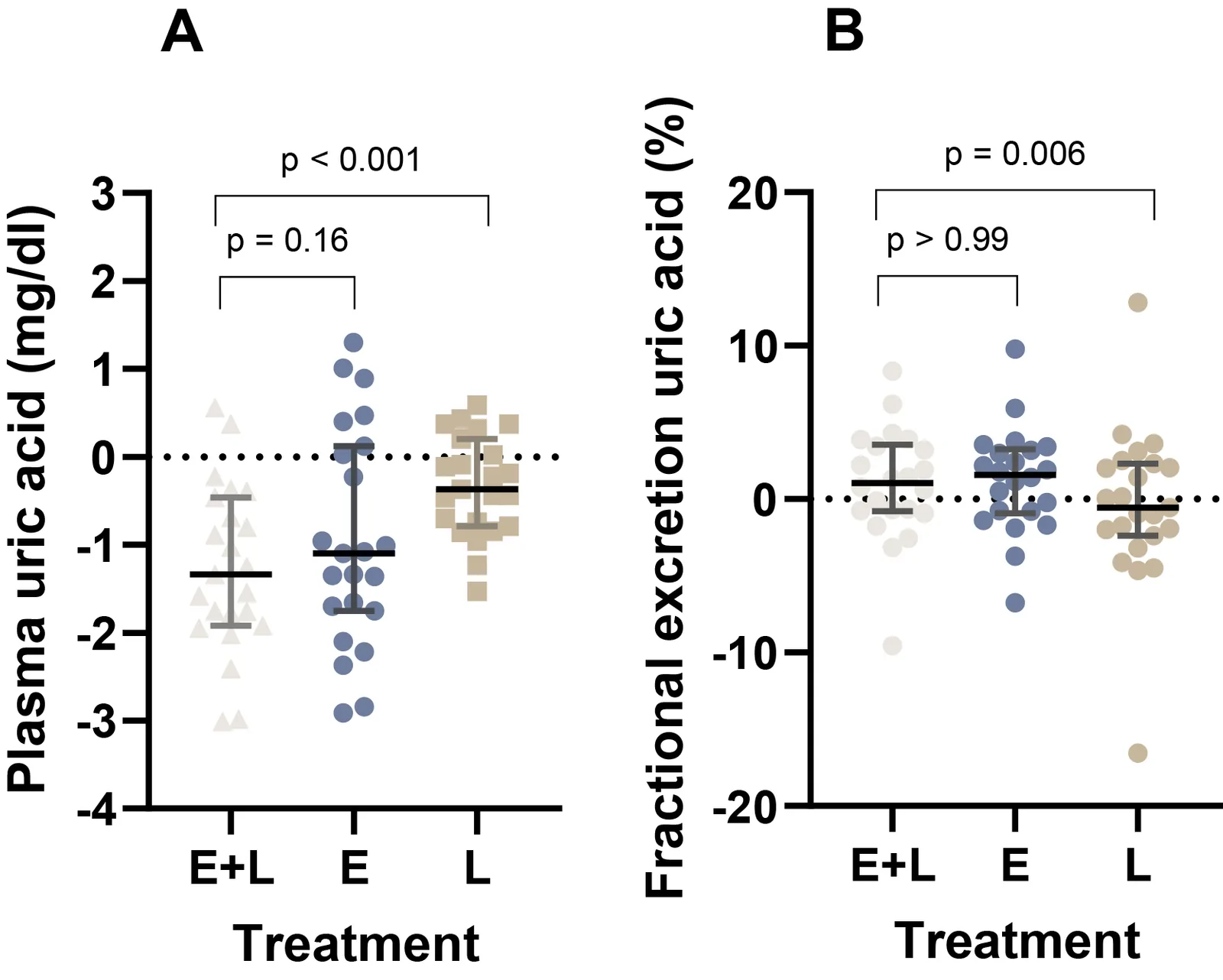
Change in uric acid after treatment compared with placebo. Data points represent the median with standard error of the mean. (A) Plasma uric acid; combination treatment versus monotherapy treatment. (B) Fractional excretion of uric acid; combination treatment versus monotherapy treatment. All analyses were corrected for multiple testing using Bonferroni correction. Abbreviations: E, empagliflozin; E + L, empagliflozin + losartan; L, losartan.

FeUA increased in all treatment arms, but none of these changes reached statistical significance (Table S2). Compared with losartan monotherapy, combination therapy increased FeUA (+1.40%; *P* = 0.006). No significant differences in FeUA were observed between monotherapy empagliflozin and combination therapy (+0.61%; *P* > 0.99) (Fig 1B).

The change in PUA after empagliflozin monotherapy was correlated with the change observed after losartan monotherapy (r = 0.58; *P* = 0.004), as well as between losartan and combination therapy (r = 0.59; *P* = 0.004) and empagliflozin and combination therapy (r = 0.91; *P* < 0.001) (Fig 2A-C). Similarly, FeUA showed correlations between each monotherapy (r = 0.62; *P* = 0.002) and the combination therapy (losartan: r = 0.78; *P* < 0.001 and empagliflozin: r = 0.91; *P* < 0.001) (Fig 2D-F).

**Figure 2:**
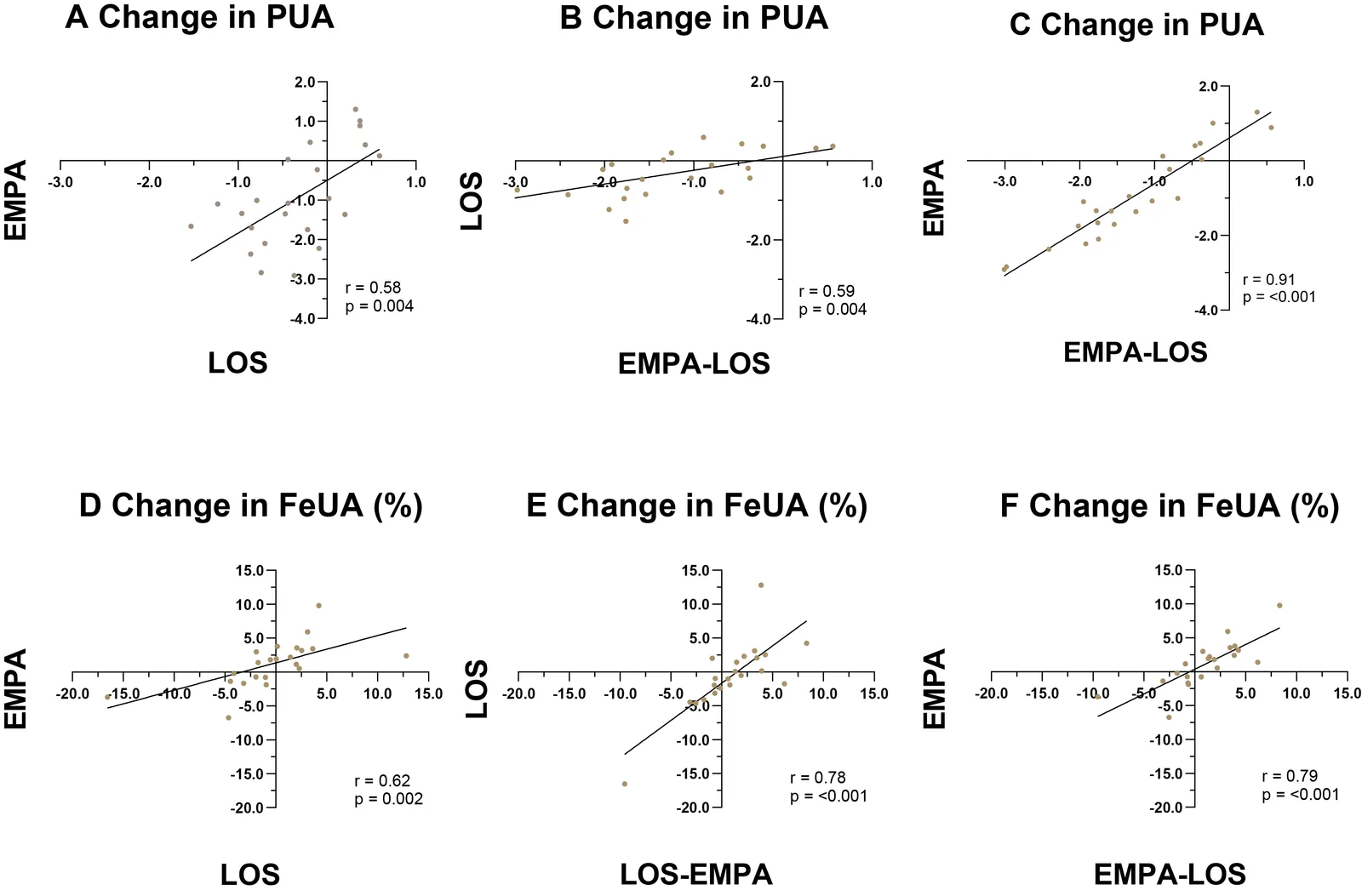
Correlation analyses of changes in PUA (mg/dL) (A-C) and FeUA (%) (D-F) among empagliflozin, losartan, and combination therapy for individuals. Abbreviations: EMPA, empagliflozin; FeUA, fractional excretion of uric acid; LOS, losartan; PUA, plasma uric acid.

No correlations were identified between changes in PUA or FeUA and changes in systolic and diastolic blood pressure, the measured glomerular filtration rate, or urinary glucose during all treatment periods.

In this post hoc analyses, empagliflozin showed reduction in PUA in contrast to losartan, which appeared to be driven by increased urinary excretion, in line with previous research.^3^ Although combination therapy reduced PUA further, this did not reach statistical significance.

Based on our previous experiments using benzbromarone, we postulated that the effects are mediated by inhibition of URAT1.^3^ Similarly, SGLT2 inhibitors were shown to promote PUA excretion via URAT1.^2^ In this study, we only observed numerical changes in FeUA. The short duration of treatment and relatively low dose of losartan may explain the lack of significant effects. Losartan has been reported to reduce URAT1 expression, which may require more prolonged therapy to induce changes in URAT1 activity.^7^ In addition, the uricosuric effect of losartan is shown to be dose dependent, with limited effects at 50 mg (Item S1).^7^ Nevertheless, we observed a strong correlation between changes in PUA and FeUA after all treatments. This suggests that empagliflozin and losartan might share a similar mechanism of action in lowering PUA. Interestingly, we observed a reduction in PUA without a corresponding increase in FeUA, suggesting the effects of empagliflozin and losartan on PUA are not solely the result of changes in excretion, and additional mechanisms are likely involved.

For example, losartan and empagliflozin affect PUA production.^8^^,^^9^ Because angiotensin II upregulates xanthine oxidase, a key enzyme in purine metabolism, losartan may lower PUA production by inhibiting this pathway (Item S1). In addition, empagliflozin reduces glucose metabolism. By increasing glycosuria and lowering overall glucose levels, purine formation may subsequently be decreased.^8^ Moreover, SGLT2 inhibitors decrease oxidative stress by downregulating NADPH oxidase activity, leading to reduced production and activation of xanthine oxidase, thereby lowering PUA.

This study has some limitations, including a small sample size, short treatment duration, low dose of losartan, and lack of dietary control (Item S1).

One of the main strengths of this study was the cross-over design. In addition, we collected 24-hour urine samples, allowing us to fully capture changes in uric acid excretion over an extended period. Furthermore, iohexol was used to measure the glomerular filtration rate, providing an accurate measurement on kidney function and renal uric acid handling.

In conclusion, our findings support the uric acid--lowering effects of empagliflozin and empagliflozin-losartan combination therapy. The reduction of PUA appears to be driven by enhanced renal excretion, although inhibition of the purine metabolism pathway may play an additional role. Extended research may be necessary to fully assess its impact.

## Supplementary Materials

Supplementary File (PDF) Figure S1; Item S1; Item S2; Tables S1 and S2.
