# Supplementary material for: Effects of Losartan, Empagliflozin, and Their Combination on Uric Acid Concentrations in People With Type 2 Diabetes
Source: Kidney Med. 2026 Jun 13;8(10):101436. doi: 10.1016/j.xkme.2026.101436 (PMC13571260; doi:10.1016/j.xkme.2026.101436)
Supplement: Supplementary file 1 [file mmc1.pdf]

Supplementary Material

Item S1: Supplementary methods

1.1 Trial design

We performed a prespecified post-hoc analysis of the RECOLAR study, a phase 4, monocenter, randomized, double-blind, placebo-controlled, four-armed crossover study assessing the renal hemodynamic effect of empagliflozin and losartan in participants with type 2 diabetes (T2D). The RECOLAR trial was conducted from September 2020 to September 2021 at the Amsterdam University Medical Centers (location VUmc) in Amsterdam, Netherlands. The study consisted of 1-week intervention period for each treatment followed by a 4-week washout period. The protocol was approved by the medical ethical review board of the VU university Medical Center (Amsterdam, the Netherlands). The study complied with the Declaration of Helsinki and Good Clinical Practice guidelines and was registered at ClinicalTrials.gov (ID: NCT04238702).

1.2 Study population

Participants were recruited from the outpatient clinic database at the Diabetes Center Amsterdam UMC. Eligible individuals included postmenopausal women and men aged 45 to 75. All participants had to have a diagnosis of type 2 diabetes and a BMI greater than 25 kg/m². Those currently being treated with metformin or sulfonylurea derivatives at a stable dose for at least three months were permitted, provided their HbA1c levels remained between 6.5% and 10.5% (48-91 mmol/mol). If patients were receiving treatment for hypertension, only beta-blockers or alpha-blockers were allowed. Exclusion criteria included any cardiovascular event in the past six months, such as acute coronary syndrome, stroke, or transient ischemic attack, a diagnosis of heart failure, rapidly

progressing or unstable kidney disease, an estimated glomerular filtration rate below 60 ml/min per 1.73 m^2, microalbuminuria, urinary retention, current urinary tract or genital infections, diabetic ketoacidosis within the last six months, or the use of non-steroidal anti-inflammatory drugs or diuretics that could not be stopped. Written consent was obtained from all participants.

1.3 Randomization and intervention

Participants were randomized to crossover sequences for treatment with empagliflozin 10mg and losartan 50mg monotherapy, combination therapy and placebo, as published previously (S7). Participants and investigators remained blinded to treatment status until database lock. Patients were instructed to take their medication once daily a 08.00 PM during complete treatment period.

1.4 End point measurements

Primary endpoints were the effect of Empagliflozin and Losartan on PUA. Secondary endpoints was the effect on FeUA, changes in systolic blood pressure, diastolic blood pressure, mGFR or urinary glucose excretion. All measurements were taken after one week of each treatment arm. The week before each clinical visit participants were instructed to a standardized sodium chloride (9-12 gram/day) diet to minimize variation in kidney physiology, checked by 24-h sodium collection. After overnight fast participants arrived at 08:00 AM at the research unit of the Diabetes Center. All measurements were performed in fasting state, semi supine position and in a temperature controlled room.

Systolic and diastolic blood pressure (SBP/DBP) and heart rate (HR) were determined by an automated device (Dinamap, GE Healthcare, UK). The mean of three measurements was used, with 1-2 minute intervals. Pulse pressure was calculated.

Stoke volume (SV) , cardiac output (CO) and systemic vascular resistance (SVR) were measured by a photoplethysmography device (Nexfin, the Netherlands).

Participants collected 24-hour urine samples to measure sodium excretion (mmol/24h), glucose (mmol/24h) and uric acid (mg/dL) levels, among others. Blood samples were drawn to measure fasting plasma glucose (mmol/L), HbA1c (%) and plasma uric (mg/dL) acid among other variables. Fractional excretion of uric (FeUA) acid was calculated using the following formula: [urine acid (urine) × creatinine(serum)] / [uric acid(serum) × creatinine(urine)] × 100%.

1.5 Statistical analyses and post-hoc outcome measures

Primary endpoints of this post-hoc analyses included PUA levels after each treatment arm. Secondary endpoints included change in FeUA systolic bloodpressure, diastolic bloodpressure, measured GFR and glycosuria. This post-hoc analysis was based on the RECOLAR study, which was originally powered for renal hemodynamic outcomes; therefore, no separate power calculation was performed for uric acid parameters. The statistical analysis was conducted on the per-protocol population using IBM SPSS 28 software (IBM, SPSS, IL), with a significance set at 0.05. To compare within groups, repeated measures ANOVA was performed in case of normal distribution. In case of non-normally distributed variables Friedman test was performed, followed by Wilcoxon signed rank tests for pairwise comparisons including 95% confidence intervals. To adjust the probability values and avoid type I error, we performed the Bonferroni correction for multiple testing. Graphs were created using GraphPad Prism software.

Supplementary references

S1. Johnson RJ, Bakris GL, Borghi C, et al. Hyperuricemia, acute and chronic kidney disease, hypertension, and cardiovascular disease: Report of a scientific workshop organized by the National Kidney Foundation. *Am J Kidney Dis*. 2018;71:851-865. DOI: 10.1053/j.ajkd.2017.12.009

S2. Badve SV, Pascoe EM, Tiku A, et al. Effects of allopurinol on the progression of chronic kidney disease. *N Engl J Med*. 2020;382(26):2504-2513. DOI: 10.1056/NEJMoa1915833

S3. Miao Y, Ottenbros SA, Laverman GD, et al. Effect of a reduction in uric acid on renal outcomes during losartan treatment: a post hoc analysis of the reduction of endpoints in non-insulin-dependent diabetes mellitus with the Angiotensin II Antagonist Losartan Trial. *Hypertension*. 2011;58(1):2-7. DOI: 10.1161/HYPERTENSIONAHA.111.171488

S4. Wolff, M. L., Cruz, J. L., Vanderman, A. J., & Brown, J. N. (2015). The effect of angiotensin II receptor blockers on hyperuricemia. *Therapeutic Advances in Chronic Disease*, *6*(6), 339-346.

S5. Smink PA, Bakker SJ, Laverman GD, et al. An initial reduction in serum uric acid during angiotensin receptor blocker treatment is associated with cardiovascular protection: a post-hoc analysis of the RENAAL and IDNT trials. *J Hypertens*. 2012;30(5):1022-1028. DOI: 10.1097/HJH.0b013e32835200f9

S6. Ferreira JP, Zannad F, Kiernan MS, et al. High-versus low-dose losartan and uric acid: An analysis from HEAAL. *J Cardiol*. 2023;82(1):57-61. DOI: 10.1016/j.jjcc.2023.04.005

S7. Landmesser U, Spiekermann S, Preuss C, et al. Angiotensin II induces endothelial xanthine oxidase activation: role for endothelial dysfunction in patients with coronary disease. *Arterioscler Thromb Vasc Biol*. 2007;27(4):943-948. DOI: 10.1161/01.ATV.0000258415.32883.bf

S8. Scholtes, R. A., Hesp, A. C., Mosterd, C. M., Geurts, F., Hoorn, E. J., Touw, D. J., ... & Van Raalte, D. H. (2022). Kidney hemodynamic effects of angiotensin receptor blockade, sodium-glucose cotransporter-2 inhibition alone, and their combination: a crossover randomized trial in people with type 2 diabetes. *Circulation*, *146*(24), 1895-1897.
DOI: 10.1161/CIRCULATIONAHA.122.061033

Figure S1.

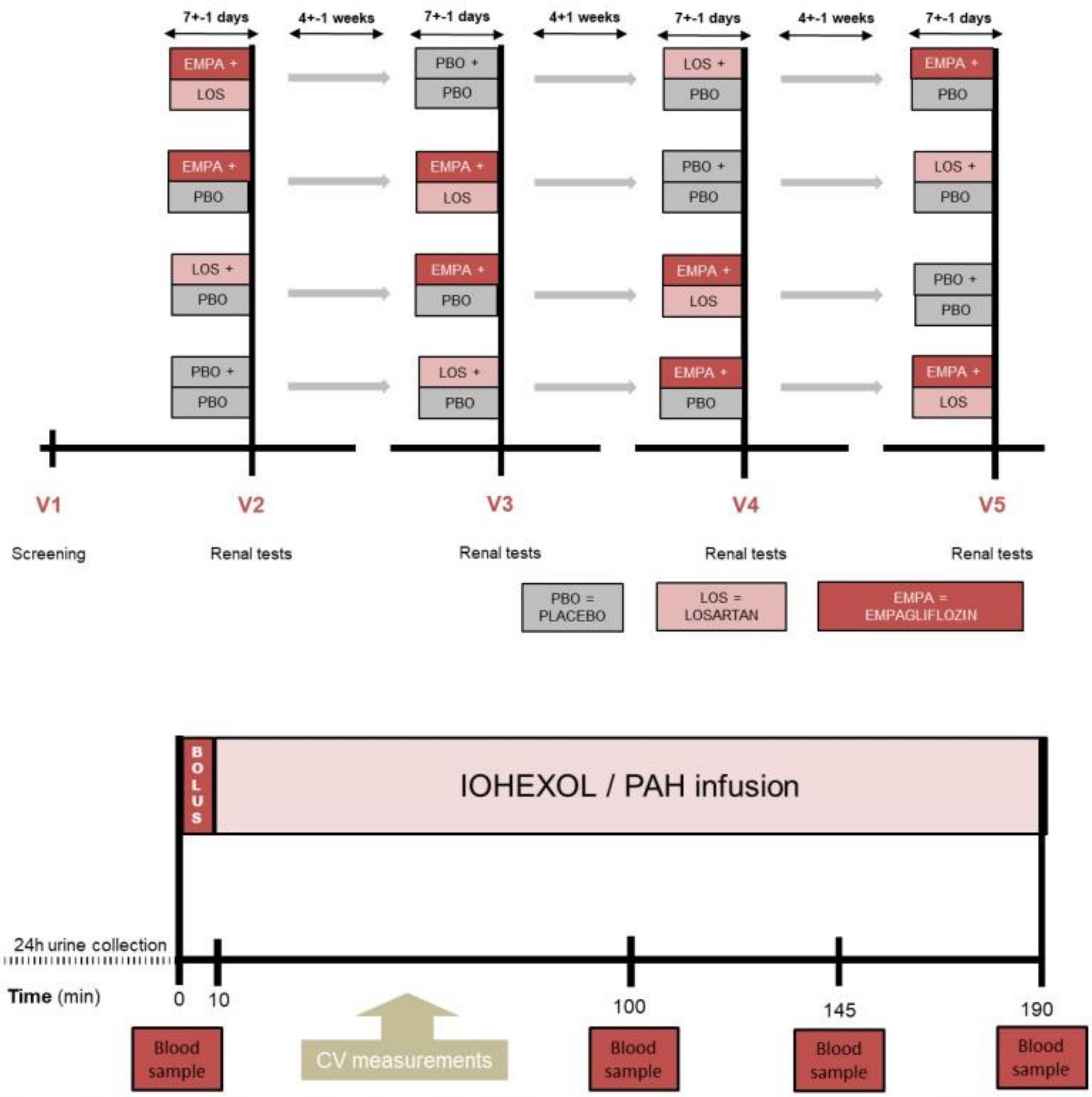

Figure S1 – (A) study design with time in weeks. (B) Schematic overview of test protocol during V2, 3, 4 and 5 with time in minutes.

Figure S1: Study design and test protocol

Table S1. Baseline characteristics

Variables	*RECOLAR, n=24*
Age	66 ± 6
Male, n (%)	21 (87.50)
BMI (kg/m2)	31 ± 3
Ethnicity–White, n (%)	24 (100)
Diabetes duration	11 (IQR 8-16)
Alcohol intake, units/week	4 (IQR 2-11)
Current smoker, n (%)	2 (8.30)
ASCVD, n (%)	3 (12.50)
Hypertension, n (%)	14 (58.30)
Systolic blood pressure, mmHg	145 ± 16
Diastolic blood pressure, mmHg	86 ± 7
HbA1c, %	7.40 ± 0.90
eGFR (CKD-EPI), ml/min/1.73m2	89.0 (IQR 79-90)
Medication use	
Metformin, n (%)	24 (100)
SU, n (%)	11 (45.80)
Statin, n (%)	17 (70.80)
Beta blocker, n (%)	2 (8.30)
Both alpha and beta blocker, n (%)	5 (20.80)
Plasma uric acid (mg/dL)	5.43 (IQR 4.91-6.56)
Fractional excretion uric acid (%)	5.10 (IQR 3.59-6.76)

Data are represented as mean ± SD, median (IQR) or frequency (%). Abbreviations: ASCVD, atherosclerotic cardiovascular disease; BMI, body mass index; CKD-EPI, Chronic Kidney Disease Epidemiology Collaboration; eGFR, estimated glomerular filtration rate; SU, sulphonyl urea derivative.

	Placebo	Empagliflozin	Δ EMP vs PLC	p-value	Losartan	Δ LOS vs PLC	p-value	Combination	Δ COM vs PLC	p-value
**PUA (mg/dL)**	5.43±1.1	4.59±1.18	-0.84	p = 0.009	4.99±1.12	-0.44	p >0.99	4.32±0.95	-1.11	p <0.001
**FeUA (%)**	5.10±4.6	6.10±3.70	+1.03	p = 0.72	5.40±3.50	+0.24	p >0.99	6.80±2.80	+1.60	p = 0.56

Table S2. Uric acid lowering after treatment compared to placebo

* Abbreviations: EMP (empagliflozin), PLC (placebo), LOS (losartan). Bonferroni correction was applied for multiple testing.
